# Unequal Access to Testing and Vaccination Services for the Homeless and Undocumented Population During COVID-19 Pandemic

**DOI:** 10.3389/ijph.2022.1604851

**Published:** 2022-06-14

**Authors:** Aldo Morrone, Anna Rita Buonomini, Alessandra Sannella, Fulvia Pimpinelli, Arianna Rotulo

**Affiliations:** ^1^ San Gallicano Dermatological Institute, Hospital Physiotherapy Institutes (IRCCS), Rome, Italy; ^2^ Human, Social, and Health Sciences Department, University of Cassino, Cassino, Italy; ^3^ Wolfson Institute of Population Health, School of Medicine and Dentistry, Queen Mary University of London, London, United Kingdom; ^4^ Department of Sustainable Health, Campus Fryslan, University of Groningen, Groningen, Netherlands

**Keywords:** access to health care, inequalities, homelessness, inequities, COVID-19, healthcare delivery, equity

## Abstract

**Objectives:** To furnish a model to ensure access and use of healthcare services to the undocumented and homeless population.

**Methods:** Between March 2020 and October 2021, public and third sector actors in Rome implemented an accessible COVID-19 screening service and vaccination program targeting the homeless and undocumented population.

**Results:** 95.6% of the patients tested negative to both rapid and molecular tests. 0.9% tested positive to both. 0.7% were false negatives, while 2.8% were false positives. None of the participants refused the diagnostic treatment. From July to October 2021, 1384 people received a complete cycle of the COVID-19 vaccine through the program. 632 (45.6%) also agreed to perform the antibodies testing before inoculation. 318 (50.31%) of these were positive at the time of vaccination.

**Conclusion:** We present a cost-effective model for reducing structural barriers to access diagnostic and preventive services for the homeless and undocumented population that can be applied to different public health settings.

## Introduction

Since its inception, social and health inequalities and inequities have characterised the current public health emergency. The assumption that SARS-Cov2 could be an equaliser between the rich and the poor has been swiftly replaced by the certainty that disparities during a pandemic can only increase [1]. Among all, the homeless and the undocumented population constitute one of the most vulnerable segments of society: social and material deprivation, unhealthy lifestyles, co-morbidities, and difficulties to enter the healthcare system make them the most exposed group to public health threats [2, 3].

In 2014, the Italian Statistical Bureau (ISTAT) estimated 50724 homeless in Italy [4]. According to a report from the pastoral charity *CARITAS Italiana*, these are mostly single, non-native men, the majority of which lives in Lombardy (30.4%), Emilia-Romagna (19.6%), and Lazio (9.2%) [5]. An investigation in Italian counselling centres suggests that almost one out of three homeless (7484 out of 26078) is between 18 and 34 years old, while 23% has between 35 and 44 years old [6]. In 2019, the Citizen Observatory on Social Marginalisation estimated that at least 21000 people had received support related to immigration and homelessness from the Social Policy Department of the Municipality of Rome [6]. Official estimates on the density of undocumented people in the country remain unknown.

Lack of access to healthcare for the homeless and the undocumented population is a well-known concern [7–10]. The situation has deteriorated since the start of the pandemic. Saturated and nearly collapsing health services have exacerbated barriers to entry to non-COVID services, neglecting treatment of pre-existing health conditions [11]. For many, bureaucratic and organisational obstacles have made COVID-19 screening and diagnostic procedures hard to reach [12].

The Italian healthcare system is heavily decentralised [13]. Accordingly, the country’s pandemic response has been fragmented and inappropriate to the needs of the vulnerable population [14]. Since 2020, for example, every region has been responsible for the organisation, management, and provision of essential epidemiological surveillance and prevention in the context of COVID-19 [15]. In the case of Lazio Region, antigen diagnostic tests (ADT) and molecular tests (PCR) are available both at the public and private levels, in the latter case, for a fee (between €15 and €60, depending on the type of swab). Access to publicly provided tests is free. However, it is contingent on an e-prescription from the general practitioner, which can be granted only to users with a social security number and proof of residence [16].

Similarly, the Italian vaccination strategy consists of each region setting up its vaccination criteria and booking systems according to their agenda settings and priorities. Besides being highly inequitable in mode and scope, until recently, none of the regional booking systems allowed access to vaccines to people without an Italian social security number [17]. This includes foreigners (e.g., tourists, migrants), undocumented people, and people without proof of residence.

To limit the social, health, and healthcare disparity gap posed by the abovementioned social and bureaucratic barriers and to decrease the risk of epidemic outbreaks among socially vulnerable segments of the population, the San Gallicano Dermatological Institute of Rome has been a central actor in the provision of COVID-19 screening and vaccines to the homeless and the undocumented population in reception centres in Rome. As soon as vaccine doses became available, the Institute promoted a successful vaccination program to population with health vulnerability (e.g., oncologic patients, psoriasis patients treated with biologic drugs) [18, 19] in addition to people with socio-economic vulnerabilities.

The purpose of this article is twofold: 1) to provide an epidemiological snapshot of a sample of the homeless and undocumented population resulting from a survey carried out in the city of Rome, Italy; 2) to furnish national health systems with a sustainable model [20] to improve access to care for the vulnerable population, in contexts of public health emergencies, in line with UN Agenda 2030 and Sustainable Development Goals n.3 and n.10 that no one is left behind.

## Methods

### COVID-19 Screening Program

In March 2020, the San Gallicano Dermatological Institute for Research and Care of Rome organised and carried out a COVID-19 screening service in three areas of Rome. The research has been authorised by the ethical committee of the National Institute for Infectious Diseases Lazzaro Spallanzani (approval of the committee n. 134). Screenings took place at the Apostolic Charity outpatient clinic (managed by Medicina Solidale Onlus—Social Medicine NGO), at the *CARITAS* outpatient facility, and at the reception centre of the not-for-profit organisation *Binario95* (Platform95).

Access to the service in the two outpatient clinics was on a walk-in basis, whereas *Binario95* set up an online booking system that users—or social workers on their behalf—could use to reserve a slot. All three facilities were equipped with social workers, nurses, and doctors. Patients were asked to sign an informed consent provided in multiple languages. The process was facilitated by cultural mediators. None of the participants refused to take part to the screening.

Social workers at the outpatient facilities were responsible for collecting users’ data and contact details upon arrival. In contrast, users at *Binario95* were given a pre-compiled module with the details entered upon online registration. Nurses and doctors—adequately equipped with the necessary personal protective equipment– were ultimately responsible for carrying out the swab test.

The screening process consisted of nasopharyngeal antigenic diagnostic tests (ADT) and a molecular swab test (PCR). Outcomes of ADTs were communicated to users after 20 min. Molecular swabs were analysed at the San Gallicano Research Institute Department of Virology using Real-Time-Polymerase Chain Reaction (PCR). Results were released to patients by email or to the facility where the testing took place (according to privacy regulations). In other cases, they were handed out at the testing location upon appointment after 24–48 h.

The surveillance path for homeless and undocumented people was the following: transfer to a COVID Hotel was immediately arranged for patients with a positive ADT (asymptomatic or paucisymptomatic) and isolation was required until receipt of the PCR test result to confirm or dismiss the ADT’s outcome. Isolation in COVID Hotels was confirmed for patients with a positive PCR for 14 days or until a negative swab test.

**FIGURE 1:**
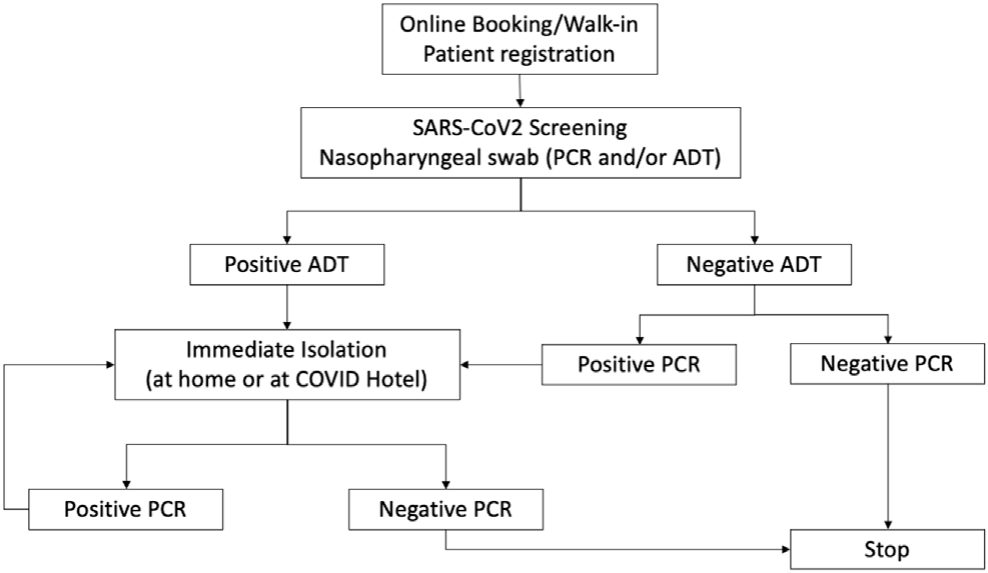
Model for COVID-19 screening of the undocumented and homeless population (Unequal Access for Homeless-Undocumented, Rome, Italy. 2021).

In the case of a negative PCR result after a positive ADT (false positive), isolation was interrupted (Figure 1). It is worth mentioning that with a negative test, the patient could access essential services such as canteens and dormitories.

False negatives, i.e., negative ADT and positive PCR, were identified after 24–48 h, dependent on laboratory timings. In this case, patients were traced and hosted in COVID Hotels. To minimise the risk of epidemic outbreaks, screening was also offered to social and health workers, which had to isolate at home in case of positive ADT and/or PCR.

### COVID-19 Vaccination Program

Starting from June 2021, the San Gallicano Dermatological Institute devoted part of their allocated COVID-19 vaccine doses to immunise Rome’s homeless population. The research has been authorised by the ethical committee of the National Institute for Infectious Diseases Lazzaro Spallanzani (approval of the committee no. 168).

Any voluntary, not-for-profit organisation or individual patients could book a vaccination slot through the *Binario95* website. Contrarily to regional vaccination booking platforms, the online form did not require users’ documents (e.g., Social Insurance Number; Italian document; proof of residence) to reserve a slot. Upon booking, patients were asked to provide their medical history to assess any contraindication to administering a particular type of vaccine. Patients were asked to sign an informed consent provided in multiple languages. The process was facilitated by cultural mediators. None of the participants refused to take part to the vaccination program.

**FIGURE 2:**
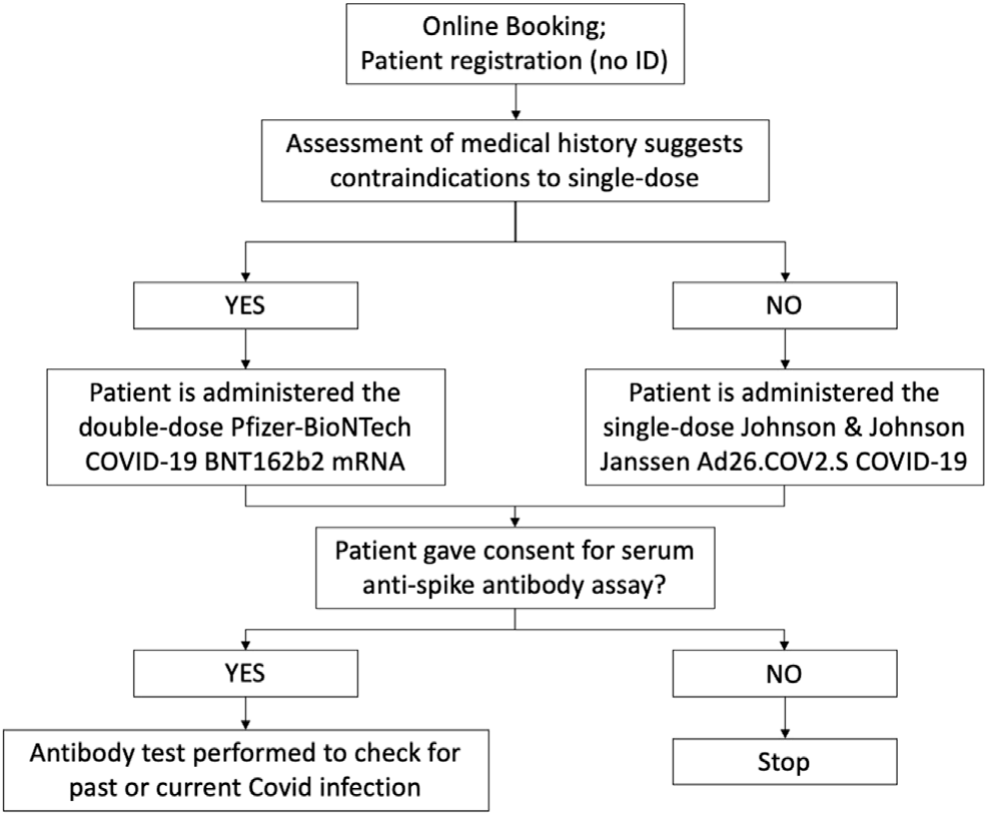
Model for COVID-19 vaccinations to the undocumented and homeless population (Unequal Access for Homeless-Undocumented, Rome, Italy. 2021).

A single-dose vaccine (Janssen Ad26.COV2.S COVID-19) was preferred to ensure patients received full immunisation without the risk of them skipping the second dose. However, the two-doses Pfizer-BioNTech COVID-19 BNT162b2 mRNA vaccine was given to patients who had contraindications to Janssen. Contingent to their consent, patients were asked to take part to a serum anti-spike antibody assay screening (Figure 2). The blood sample were obtained voluntarily through venepuncture at the time of vaccination. IgG antibodies targeting the S1 and S2 domains of SARS-CoV-2 spike protein were tested in serum samples using a commercially available capture chemiluminescence immunoassay (CLIA) kit (LIAISON (R) SARS-CoV-2 S1/S2 IgG DiaSorin, Sallugia Italy). The employed test identifies the sample as positive with a value greater than 15 AU/ml.

## Results

The data below refers to the COVID-19 screening between March 2020 and October 2021. Within this timeframe, 6468 tests were performed for the detection of SARS-CoV2. Results of 33.67% (2178) of the total PCR and ADT tests were registered online through *Binario95*’s data platform.

Recipients had a median age of 40 years old, ranging from 1 to 85 years old. 5.7% (368) were minors (aged below 18 years old); 5.4% (348) were over-65; 29.9% (1932) were young adults between 18 and 34 years old; 34% (2198) were people aged between 35 and 49; 24.6% (1590) were patients aged between 50 and 65. Information was not available for 0.5% of patients. 43.9% (3616) of users were cisgender women, and 55.9% (2840) were cisgender men. 0.2% [32] were MtoF transgender. Nationality of patients was Italian for 57.3% (3707), whereas 7.1% (459) were EU citizens, and 35.6% (2302) were non-EU nationals (Table 1).

**TABLE 1:** Characteristics of the screened population (Unequal Access for Homeless-Undocumented, Rome, Italy. 2021).

Recipients’ characteristics		N (%)
Age brackets (median 40; range 1–85)	<18 years	368 (5.7)
	18–34	1932 (29.9)
	35–49	2198 (34.0)
	50–65	1590 (24.6)
	>65	348 (5.4)
	not reported	32 (0.5)
Gender	Cis men	3616 (55.9)
	Cis women	2840 (43.9)
	Transgender Women	12 (0.2)
Country/area of origin	Italy	3707 (57.3)
	EU Member State	459 (7.1)
	Non-EU Country	2302 (35.6)

Considering the results of molecular swab tests, 242 positives were identified (3.7%).

Comparing the results of two methods: 95.6% (2082) of patients screened with both tests were negative (i.e., negative ADT and negative PCR test). 0.9% [20] tested positive to both ADT and PCR tests. 0.7% [16] were false negatives, i.e., had a negative ADT dismissed by a positive PCR test, while 2.8% (60) were false positives, that is, patients with a positive ADT and a negative PCR. The overall concordance is 96.5% (Table 2).

**TABLE 2:** ADT/PCR concordance (Unequal Access for Homeless-Undocumented, Rome, Italy. 2021).

	Negative PCR	Positive PCR
Negative ADT	2082 (95.6%)	16 (0.7%)
Positive ADT	60 (2.8%)	20 (0.9%)
Concordance: 2102 (96.5%)		

From July to October 2021, 1384 homeless and undocumented people received a complete cycle of the COVID-19 vaccine through the program. A single-dose vaccine (Janssen Ad26.COV2.S COVID-19) was preferred to ensure patients received full immunisation without the risk of missing the second dose. When patients had contraindications to Janssen, the two-doses Pfizer-BioNTech COVID-19 BNT162b2 mRNA vaccine was given.

Overall, 1090 (78.8%) persons received a single-dose vaccine: 39.4% (383) cisgender women, 1.5% [15] MtoF transgender, and 59.1% (573) cisgender men. Other 294 (21.2%) persons received two-doses of Pfizer-BioNTech vaccine: 119 (45.8%) were cisgender women, 6 (2.3%) MtoF transgender, and 135 (51.9%) cisgender men.

632 (45.6%) of these also agreed to perform the serum assay of anti-spike antibodies before inoculation. 48.5% (306) were cisgender women, and 51.5% (326) were cisgender men, with a mean age of 39.4 years, a median of 37 years old, and a range between 14–81 years old. The mean age for women was slightly higher than for men (40.3 and 38.6, respectively). 318 patients (50.31%) were positive at the time of vaccination: 129 (40.5%) were cisgender women, and 189 (59.5%) were cisgender men. The mean age was 39.5 years.

When compared to a control group, the percentage is significantly higher (4.1% in healthcare workers and 4.8% in over 80 people), as reported by Pellini et al.[21].

## Discussion

The COVID-19 pandemic has tragically impacted the most vulnerable fringes, including the homeless population. Barriers to access coupled with high healthcare needs were already a pressing issue among the community [1]. Lack of shelter and basic sanitation, unhealthy lifestyles, pre-existing health conditions, and chronic degenerative pathologies are factors that may increase the risk of severe COVID-19 illness and mortality [3, 22]. Moreover, being constantly on the move, taking on occasional jobs, sleeping in crowded shelters, and experiencing continuous low access to public healthcare services further expose these categories to the risk of infection and spread [11]. These aspects have transformed the current pandemic into a syndemic, which has worsened social and economic inequalities, with an impact on individual rights. This has caused further impoverishment of people in a state of vulnerability and/or poverty, with effects on the state of health.

Although solidarity mechanisms were activated rather quickly during the first pandemic wave, the current emergency has revealed the “infinite variations of differences” between societies, nations, continents, which ultimately weight on the most fragile people [23]. Against this backdrop, the current crisis is not just a health crisis but an economic and social one.

Preventing and controlling epidemic outbreaks in large metropolitan cities has become an urgent challenge. A step to effectively achieve so is to enforce active screening and access to diagnostic and preventive tools to the whole population, with a keen eye on the most vulnerable and needy people, i.e., the homeless and the undocumented. This study presents a model for providing testing and vaccines to those who face structural barriers to access healthcare in Rome, Italy. There are two interpretations for the positive rate for anti-spike antibodies (50.31%) among homeless and undocumented people. First, the study population—and especially the homeless one—may have been more vulnerable to SARS-CoV2 due to the low levels of sanitation and crowded housing conditions they are exposed to. Another explanation for the higher seropositivity is that this segment of the population of the study group was reached later in the vaccination campaign than the rest of population, especially when compared to the elderly population and healthcare workers of the control group, who were the first to be vaccinated against SARS-CoV-2 in line with government regulations.

In the Italian case, the system’s fragmentation has meant a total lack of support to the vulnerable categories as the pandemic gained ground [24]. For example, people affected by COVID-19 had the legal obligation to isolate themselves. This, however, requires housing that is not available to some segments of society. The third sector stepped in to reserve some shelters to receive positive cases among the homeless and the undocumented, with social workers being regularly tested to contain the spread [25]. Similarly, the welfare system requisitioned private hotels to transform into medical accommodations for homeless patients, a costly alternative that can be financially sustainable only for short periods [26, 27].

Moreover, by national regulations, shelter access is contingent to proof of a negative ADT [28]. However, paying a private provider remains the only option when users cannot access free public tests due to structural and bureaucratic barriers. High levels of poverty and material deprivation can prevent people from getting tested. The consequence is serious for the system too. Epidemiological surveillance and treatment can be complicated to supply to homeless and undocumented people. Failure to provide these services effectively can affect the entire community [29]. As suggested by Barbieri, an active screening and surveillance program among the homeless population is essential [30]. A nationwide, rather than a local-based approach to tracing and surveillance is necessary to identify positive cases and provide them with the necessary healthcare [29]. Most importantly, interventions of such should be guaranteed by the NHS, with the third sector playing a marginal rather than a central role.

Homeless and undocumented people seem to be more at risk of contracting COVID-19. This could directly affect low vaccination rates among this population segment for the reasons mentioned above. However, several structural barriers impede the homeless and the undocumented population from accessing COVID-19 vaccines [17]. The vaccination plan in 2021 was prepared in agreement with the Regional Health Authority of Lazio and allowed patients without direct access to the INHS to receive immunisation. Although laudable, the model—in its current form—is but a patch in the hole of the healthcare system. Provision of vaccines should be as decentralised as possible to favour the reachability of the service [31]. However, leaving the organisational responsibility to the third sector and local providers alone is not a feasible long-term solution since attaining a comprehensive vaccination distribution and coverage diverges from the main tasks of local and private not-for-profit providers [14]. According to Ralli-Morrone et al. [32], early identification of asymptomatic carriers is crucial in relatively unsafe settings like homeless shelters, where infections can easily spread and cause outbreaks with serious consequences for individuals and public health.

Studies on interventions for the homeless and the undocumented population are not frequent and their reporting in the context of a pandemic is scarce. This work described the epidemiological prevention of COVID-19 pandemic in Italy, using clearly defined target groups, with a focus on the vulnerable segments of the population. The study is embedded within a theoretical framework of social deprivation, as a first step in filling the mentioned research gap.

Despite the efforts in producing a robust contribution, this work suffers of some limitations. Replicability of the study is limited in that the research question depends on the pandemic and the level of access to care depends on the specific structure of the health system of interest. Future contributions should compare access to care for the homeless and undocumented population in different healthcare settings. Furthermore, the study may suffer from participants’ bias, in that participants in this study are expected to be those truly in need of testing and vaccination. Similarly, due to regional emergency regulations, the control group employed for the study has been recruited among the patients and staff of the San Gallicano Hospital, whose demographic and health characteristics may only partially match those of the study population. Future similar studies should aim at recruiting a control population with similar characteristics of those of the study group. Another weakness is the geographically narrow focus of the study. Regional health systems in other Italian areas, as well as different health systems in other country contexts may have undergone changes during the pandemic emergency that were not applied to the health system of study. To ensure the generalisability of our observations, future research should test our findings against in different national or international settings. Lastly, the control group employed for the study.

The proposed model shows that ensuring access to diagnostic and preventive services to patients without documents can be achieved cost-effectively. Most importantly, the proposed interventions can contribute to achieve the UN’s sustainable development goals for health equity, for poverty reduction, and for fighting discriminations. Although the third sector and local healthcare facilities have stepped up to ensure provision to all, it is State’s responsibility to guarantee fair provision and access continue, in the aim of minimising the arising of continuous public health risks.

## References

[1] Bambra C Riordan R Ford J Matthews F. The COVID-19 Pandemic and Health Inequalities. J Epidemiol Community Health (2020). 74:964–8. 10.1136/jech-2020-214401 32535550 PMC7298201

[2] Kondilis E Papamichail D McCann S Orcutt M Carruthers E Veizis A The Impact of the COVID-19 Pandemic on Migrants, Refugees and Asylum Seekers in Greece: A Retrospective Analysis of National Surveillance Data. SSRN Electron J (2021). 2021. 10.2139/ssrn.3788086 PMC8256175 34258570

[3] Ralli M Cedola C Urbano S Morrone A Ercoli L. Homeless Persons and Migrants in Precarious Housing Conditions and COVID-19 Pandemic: Peculiarities and Prevention Strategies. Eur Rev Med Pharmacol Sci [Internet] (2020). 24:9765–7. 10.26355/eurrev_202009_23071 33015824

[4] ISTAT ISB. Le Persone Senza Dimora, Year 2014 - Homeless People, Year 2014. Rome, Italy: Istat (2015).

[5] Caritas Italiana. Vasi Comunicanti: Report on Poverty and Social Deprivation in Italy and at the Gateway of Europe. Rome, Italy: Caritas Italiana (2016).

[6] Lelo K Monni S Radicchi A Tomassi F. La Mappa degli invisibili: migranti e persone senza dimora - the map of invisibles: migrants and homeless people. Rome, Italy: Binario95 (2022).

[7] Beck TL Le T-K Henry-Okafor Q Shah MK. Medical Care for Undocumented Immigrants. Physician Assist Clin (2019). 4:33–45. 10.1016/j.cpha.2018.08.002 32289088 PMC7141175

[8] Legido-Quigley H Pocock N Tan ST Pajin L Suphanchaimat R Wickramage K Healthcare Is Not Universal if Undocumented Migrants Are Excluded. BMJ (2019). 366:l4160. 10.1136/bmj.l4160 31527060 PMC6741752

[9] Mona H Andersson LMC Hjern A Ascher H. Barriers to Accessing Health Care Among Undocumented Migrants in Sweden - a Principal Component Analysis. BMC Health Serv Res (2021). 21:830. 10.1186/s12913-021-06837-y 34404416 PMC8369752

[10] Baggett TP O’Connell JJ Singer DE Rigotti NA. The Unmet Health Care Needs of Homeless Adults: A National Study. Am J Public Health (2010). 100:1326–33. 10.2105/AJPH.2009.180109 20466953 PMC2882397

[11] Albon D Soper M Haro A. Potential Implications of the COVID-19 Pandemic on the Homeless Population. Chest (2020). 158:477–8. 10.1016/j.chest.2020.03.057 32389724 PMC7205705

[12] Liu M Hwang SW. Health Care for Homeless People. Nat Rev Dis Primer (2021). 7:5. 10.1038/s41572-020-00241-2 33446661

[13] Rotulo A Paraskevopoulou C Kondilis E. The Effects of Health Sector Fiscal Decentralisation on Availability, Accessibility, and Utilisation of Healthcare Services: A Panel Data Analysis. Int J Health Pol Manag (2021). 2021. 10.34172/ijhpm.2021.163 PMC9818112 35021611

[14] Ciarini A Neri S. 'Intended' and 'unintended' Consequences of the Privatisation of Health and Social Care Systems in Italy in Light of the Pandemic. Transfer: Eur Rev Labour Res (2021). 27(3):303–17. 10.1177/10242589211028458

[15] Istituto Superiore di Sanità. COVID-19 Integrated Surveillance Data in Italy (2022). Available from: https://www.epicentro.iss.it/en/coronavirus/sars-cov-2-dashboard.

[16] Lazio R. Test per il Coronavirus e dove farli - Coronavirus tests and where to get them (2022). Available from: https://www.salutelazio.it/covid-19-l-offerta-di-test-nel-lazio.

[17] Armocida B Formenti B Missoni E D’Apice C Marchese V Calvi M Challenges in the Equitable Access to COVID-19 Vaccines for Migrant Populations in Europe. Lancet Reg Health - Eur (2021). 6:100147. 10.1016/j.lanepe.2021.100147 34124708 PMC8179687

[18] Di Noia V Pimpinelli F Renna D Barberi V Maccallini MT Gariazzo L Immunogenicity and Safety of COVID-19 Vaccine BNT162b2 for Patients with Solid Cancer: A Large Cohort Prospective Study from a Single Institution. Clin Cancer Res (2021). 27:6815–23. 10.1158/1078-0432.ccr-21-2439 34583970

[19] Cristaudo A Graceffa D Pimpinelli F Sperati F Spoletini G Bonifati C Immunogenicity and Safety of anti‐SARS‐CoV‐2 BNT162b2 Vaccine in Psoriasis Patients Treated with Biologic Drugs. J Eur Acad Dermatol Venereol (2022). 36:e266–e268. 10.1111/jdv.17861 34897821

[20] Nocenzi M Sannella A. Perspectives for a New Social Theory of Sustainability. Berlin, Germany: Springer Nature (2021).

[21] Pellini R Venuti A Pimpinelli F Abril E Blandino G Campo F Early Onset of SARS-COV-2 Antibodies after First Dose of BNT162b2: Correlation with Age, Gender and BMI. Vaccines (2021). 9:685. 10.3390/vaccines9070685 34206312 PMC8310011

[22] Tan LF Chua JW. Protecting the Homeless during the COVID-19 Pandemic. Chest (2020). 158:1341–2. 10.1016/j.chest.2020.05.577 32531225 PMC7283054

[23] Morrone A. Covid-19 fra mito e realtà. Rome, Italy: Armando Editore (2021).

[24] Bonizzoni P Dotsey S. Migration and Legal Precarity in the Time of Pandemic: Qualitative Research on the Italian Case. Two Homel (2021). 54:117–30. 10.3986/dd

[25] Mirisola C, ed. Indicazioni operative ad interim per la gestione di strutture con persone ad elevata fragilità e marginalità socio-sanitaria nel quadro dell’epidemia di covid-19 - Interim operational indications for the management of structures with people with high socio-health fragility and marginalization in the context of the covid-19 epidemic. Rome, Italy: NIHMP - National Institute for Health Migration and Poverty (2020). [Internet].

[26] MacKenzie OW Trimbur MC Vanjani R. An Isolation Hotel for People Experiencing Homelessness. N Engl J Med (2020). 383:e41. 10.1056/NEJMc2022860 32706951 PMC7397556

[27] Fuchs JD Carter HC Evans J Graham-Squire D Imbert E Bloome J Assessment of a Hotel-Based COVID-19 Isolation and Quarantine Strategy for Persons Experiencing Homelessness. JAMA Netw Open (2021). 4:e210490. 10.1001/jamanetworkopen.2021.0490 33651111 PMC7926291

[28] Camera dei Deputati (High Chamber). Health Measures to Fight the Coronavirus Emergency - Misure Sanitarie Per Fronteggiare L’emergenza Coronavirus (2021). Available from: https://www.camera.it/temiap/documentazione/temi/pdf/1214749.pdf?_1605385480691.

[29] Di Simone E De Leo A Panattoni N Bonfà F Tatangelo M Tallarita V COVID-19 Detection and Spread Control: what Initiatives in Italy for the Homeless Population? Eur Rev Med Pharmacol Sci (2022). 26:340–4. 10.26355/eurrev_202201_27785 35049012

[30] Barbieri A. CoViD-19 in Italy: Homeless Population Needs protection. Recenti Prog Med (2020). 111:295–6. 10.1701/3366.33409 32448878

[31] Rotulo A Epstein M Kondilis E. Fiscal Federalism vs Fiscal Decentralization in Healthcare: a Conceptual Framework. Hippokratia (2020). 24(3):107–13. 34239287 PMC8256788

[32] Ralli M Morrone A Arcangeli A Ercoli L. Asymptomatic Patients as a Source of Transmission of COVID-19 in Homeless Shelters. Int J Infect Dis (2021). 103:243–5. 10.1016/j.ijid.2020.12.031 33321208 PMC7834802

